# Immunodynamics of Th17 cells in HIV-1 subtype ‘C’ infection

**DOI:** 10.1186/1471-2334-12-S1-O3

**Published:** 2012-05-04

**Authors:** Madhu Vajpayee, Alpana Singh, Sharique A Ali, Neeraj Kumar Chauhan, Ravinder Singh

**Affiliations:** 1 Department of Microbiology, All India Institute of Medical Sciences, New Delhi, India

## Background

Th17 cells are IL-17 producing CD4-T cells which play a vital role in inflammatory responses, antimicrobial defense and autoimmunity. However, the involvement of Th17 cells in HIV-1 infection especially in subtype-C is not yet identified. Thus through this study we try to dissect the role of Th17 cells in HIV-1 subtype ‘C’ infection.

## Methods

31 HIV seropositive antiretroviral therapy naïve and 8 HIV uninfected healthy control subjects were recruited and characterized as being early, late or slow progressor. Peripheral blood mononuclear cells were isolated from each study subject and stimulated with HIV-1 subtype ‘C’ gag peptide pool and assessed for IL-17 cytokine producing CD4-T cells using intracellular cytokine staining. All clinical groups were statistically compared by Kruskal-Wallis test and Spearman’s correlation coefficient was calculated for correlation of different variables.

## Results

Here we reported that both frequency and functionality of HIV-1 specific Th17 cells were induced in early and slow progressors but were significantly reduced (p<0.001) at late stage of infection in peripheral blood. Also a significant negative correlation (ρ=0.55; P=0.0004) was observed between HIV-1 plasma viral load and gag specific %IL-17 production via CD4-T cells.

## Conclusion

This study showcases a comprehensive picture of Th17 cellular dynamics in HIV-1 subtype-C infection. Further, our data establishes that higher frequencies of HIV specific Th17 cells correlates with better control of viral replication and can be used as immune correlate of protection.

